# The impact of cumulative obstetric complications and childhood trauma on brain volume in young people with psychotic experiences

**DOI:** 10.1038/s41380-023-02295-6

**Published:** 2023-10-30

**Authors:** Kate Merritt, Pedro Luque Laguna, Arjun Sethi, Mark Drakesmith, Sarah A. Ashley, Michael Bloomfield, Leon Fonville, Gavin Perry, Tom Lancaster, Stavros I. Dimitriadis, Stanley Zammit, C. John Evans, Glyn Lewis, Matthew J. Kempton, David E. J. Linden, Abraham Reichenberg, Derek K. Jones, Anthony S. David

**Affiliations:** 1 Division of Psychiatry, Institute of Mental Health, University College London, London, UK; 2 The Cardiff University Brain Research Imaging Centre (CUBRIC), Cardiff University, Cardiff, UK; 3 Department of Forensic & Neurodevelopmental Sciences, IOPPN, King’s College London, London, UK; 4 Invicro LLC, London, UK; 5 Department of Psychology, Bath University, Bath, UK; 6 Department of Clinical Psychology and Psychobiology, Faculty of Psychology, University of Barcelona, Passeig de la Vall d’Hebron, 171, 08035 Barcelona, Spain; 7 Bristol Medical School (PHS), University of Bristol, Bristol, UK; 8 Psychosis Studies Department, IOPPN, King’s College London, London, UK; 9 School for Mental Health and Neuroscience, Maastricht University, Maastricht, The Netherlands; 10 Icahn School of Medicine at Mount Sinai, New York, NY USA

**Keywords:** Neuroscience, Schizophrenia

## Abstract

Psychotic experiences (PEs) occur in 5–10% of the general population and are associated with exposure to childhood trauma and obstetric complications. However, the neurobiological mechanisms underlying these associations are unclear. Using the Avon Longitudinal Study of Parents and Children (ALSPAC), we studied 138 young people aged 20 with PEs (*n* = 49 suspected, *n* = 53 definite, *n* = 36 psychotic disorder) and 275 controls. Voxel-based morphometry assessed whether MRI measures of grey matter volume were associated with (i) PEs, (ii) cumulative childhood psychological trauma (weighted summary score of 6 trauma types), (iii) cumulative pre/peri-natal risk factors for psychosis (weighted summary score of 16 risk factors), and (iv) the interaction between PEs and cumulative trauma or pre/peri-natal risk. PEs were associated with smaller left posterior cingulate (*p*FWE < 0.001, *Z* = 4.19) and thalamus volumes (*p*FWE = 0.006, *Z* = 3.91). Cumulative pre/perinatal risk was associated with smaller left subgenual cingulate volume (*p*FWE < 0.001, Z = 4.54). A significant interaction between PEs and cumulative pre/perinatal risk found larger striatum (*p*FWE = 0.04, *Z* = 3.89) and smaller right insula volume extending into the supramarginal gyrus and superior temporal gyrus (*p*FWE = 0.002, *Z* = 4.79), specifically in those with definite PEs and psychotic disorder. Cumulative childhood trauma was associated with larger left dorsal striatum (*p*FWE = 0.002, Z = 3.65), right prefrontal cortex (*p*FWE < 0.001, *Z* = 4.63) and smaller left insula volume in all participants (*p*FWE = 0.03, *Z* = 3.60), and there was no interaction with PEs group. In summary, pre/peri-natal risk factors and childhood psychological trauma impact similar brain pathways, namely smaller insula and larger striatum volumes. The effect of pre/perinatal risk was greatest in those with more severe PEs, whereas effects of trauma were seen in all participants. In conclusion, environmental risk factors affect brain networks implicated in schizophrenia, which may increase an individual’s propensity to develop later psychotic disorders.

## Introduction

Psychotic experiences (PEs), such as delusions, hallucinations, and thought interference, occur in 5–10% of the general population and are associated with an increased risk of developing later mental disorders including psychosis [1]. Risk factors for psychotic disorders and PEs include exposure to adverse environments, both prenatally and during childhood, among others [2]. Exposure to prenatal and perinatal adverse events (i.e., complications during pregnancy and birth) is associated with an increased risk of developing psychosis [3] and PEs [4, 5] by around 1.5 times. Childhood psychological trauma is associated with a two to three fold increased odds for developing PEs [6, 7], three fold increased odds for psychosis [8], and more severe psychotic symptoms [9]. However, the neurobiological mechanisms underlying these associations are unclear [10], and it is not known whether these diverse environmental risk factors have common or distinct effects on the brain and how this interacts with psychosis risk.

While numerous MRI studies have examined the effect of separate environmental risk factors on the brain [11–18], epidemiological evidence suggests that the accumulation of risk factors rather than exposure to a single factor has a greater impact on schizophrenia risk [6, 19–21], and thus may have a cumulative effect on the brain. However, obtaining accurate data on multiple childhood trauma and prenatal risk factors is difficult, as birth records may not be available for large samples, and these measures are often assessed retrospectively and hence prone to bias.

A major obstetric risk factor, preterm birth, is associated with reductions in whole brain volume, and regionally in the thalamus, hippocampus and basal ganglia [14, 15, 17, 18, 22–26] as well as increases in the primary visual cortex [14, 18, 24]. The association between pre- and perinatal adversities and altered brain structure may be stronger in patients with schizophrenia, suggesting that genetic vulnerability and pre- and perinatal factors both affect neurodevelopment to increase the risk of schizophrenia. For example, perinatal hypoxia is associated with reduced grey matter volume throughout the cortex in schizophrenia patients and their non-psychotic siblings but not in controls [27]. Similarly, caudate volume was reduced in schizophrenia patients but not controls exposed to asphyxia-related obstetric complications, although other subcortical brain volumes were reduced in both patients and controls [28]. Maternal infection is also shown to affect offspring’s brain structure, with one study finding an association with lower grey matter volume in schizophrenia patients only [29], whereas another study reported this effect in both controls and patients [27]. These studies typically examine pre/perinatal risk factors in isolation, however a recent systematic review and meta-analysis identified more than 30 pre- and perinatal factors associated with an increased risk of developing psychosis [3]. Effects of hypoxia on brain volume are amplified in cases born small for their gestational age [27, 30], suggesting a cumulative effect, and the need to study pre- and perinatal risk factors in combination. To date, MRI studies of the effects of pregnancy and birth complications in those deemed to be at an increased risk for developing psychosis are generally limited to the relatives of patients, who have often passed the peak risk age for developing schizophrenia. Focusing on non-clinical populations with sub-threshold PEs may identify early biomarkers associated with the development of psychosis and other mental disorders.

In contrast to the global brain alterations seen with pre and perinatal risk factors, childhood psychological trauma may be associated with more localised brain changes in frontal and limbic networks. Meta-analyses report smaller grey matter volume in the hippocampus, amygdala, cingulate, striatum, dorsolateral prefrontal cortex and middle temporal gyrus in healthy individuals exposed to childhood psychological trauma [31–37] as well as larger volumes in superior frontal, precentral and occipital gyri [33, 36] and subcortical limbic areas [38]. It is unclear how the effects of trauma combine with genetic risk for schizophrenia [39–41]. MRI studies show a similar effect of trauma on brain structure in both those at a high risk of developing schizophrenia and controls [11, 12, 39, 42–44], with fewer studies reporting effects that are specific to high risk individuals [13, 45, 46]. Grey matter volume alterations associated with trauma are generally observed in the frontal cortex, amygdala and hippocampus, although the majority of studies use a region of interest (ROI) approach, focusing on these three regions [11, 12, 42, 44, 45]. The use of whole brain approaches would complement existing findings by providing an unbiased method to identify additional brain regions associated with psychological trauma. Experiences of psychological trauma are diverse, with some studies examining specific types of trauma such as physical or sexual abuse [11–13, 43, 45]. It is reasonable to hypothesise that the accumulation of multiple trauma types will have a greater impact on brain structure, but this approach has been adopted by few studies [39, 43, 45, 46], all of which were limited to using retrospective measures of trauma.

We sought to assess the cumulative effects of distinct environmental risk factors on the brain and later psychotic symptoms, by examining (i) a number of pre- and perinatal risk factors measured during pregnancy and at birth and (ii) later psychological trauma occurring in childhood and adolescence. We capitalised on the rich dataset from the Avon Longitudinal Study of Parents and Children (ALSPAC) population-based birth cohort, to examine a subset of participants who developed PEs as well as controls who underwent MRI scanning at age 20 (*n* = 434). This study investigates whether volumetric MRI measures of grey matter are associated with (i) PEs, (ii) cumulative childhood psychological trauma (summary score of 6 trauma types assessed prospectively at multiple timepoints from infancy to age 17 years, with trauma types weighted by their association with PEs) and (iii) cumulative pre- and perinatal risk factors for psychosis (summary score of 16 pre- and perinatal risk factors associated with psychosis [3], assessed during pregnancy and birth, weighted by their association with psychosis). Crucially we also set out to examine whether the effects of cumulative psychological trauma or pre/peri-natal risk on the brain are specific to those with PEs or are generalisable to all participants including controls, by examining the interaction between PEs status and cumulative risk.

We hypothesised that PEs would be associated with greater exposure to cumulative psychological trauma and cumulative pre/perinatal risk. Using a whole brain approach, we hypothesised that PEs would be associated with reduced volume in the frontal, cingulate and temporal cortex, as identified in high risk subjects previously [47]. We further hypothesised based on the previous literature that cumulative psychological trauma would be associated with volume alterations in frontal and limbic structures in all participants (PEs and controls), whereas cumulative pre- and perinatal risk would be associated with widespread volume reductions specific to individuals who developed PEs.

## Methods

### Sample

Participants were drawn from the UK ALSPAC [48–50], a population-based birth cohort from the South West of England, recruited in 1990–91 (http://www.bristol.ac.uk/alspac/) [48]. Written informed consent was obtained prior to scanning, and participants received financial compensation. Approval was granted by Cardiff University, the ALSPAC Ethics and Law Committee and the Local Research Ethics Committees. Study data were collected and managed using REDCap electronic data capture tools hosted at the University of Bristol [51]. REDCap (Research Electronic Data Capture) is a secure, web-based software platform designed to support data capture for research studies. The study website contains details of all the data that is available through a fully searchable data dictionary and variable search tool http://www.bristol.ac.uk/alspac/researchers/our-data/. PEs were assessed using the Psychosis-Like Symptom Interview [52] carried out at age 17–18 years and rated as (i) absent, (ii) suspected, (iii) definitely present following a semi structured interview based on the Schedules for Clinical Assessment in Neuropsychiatry version 2.0 (WHO, 1994) by a psychologist, or (iv) psychotic disorder, defined as definite PEs that occurred at least once per month over the previous 6 months and either caused severe distress, had a markedly negative impact on social or occupational function, or led to help seeking. This study combines imaging data from a study recruiting ALSPAC participants based on PEs [53–57] plus participants from an overlapping study sample recruited on the basis of polygenic risk scores [58]. Magnetic resonance imaging was carried out at age 20 years in 138 participants with PEs and 275 individuals without PEs serving as controls.

### Exposures

This study examines 2 exposures: cumulative psychological trauma and cumulative pre and perinatal risk. We selected pre- and perinatal biological risk factors that have been identified to be significantly associated with psychosis [3]. 16 risk factors (binarised) were available in the ALSPAC dataset, measured during the mothers pregnancy and at birth from the mother using self-report questionnaires, and also from data extracted from obstetric and neonatal medical records [59]. These risk factors include premature birth (<37 weeks), birth weight (<2500 g), paternal (<20 yrs and ≥35 yrs) and maternal age (30–34 yrs), maternal hypertension, pre-eclampsia, polyhydramnios, hypoxia, asphyxia (see Supplementary Table 1 for full list). For cumulative psychological trauma, measures of 6 types of trauma were reported at multiple timepoints from infancy to age 17 years (physical cruelty, domestic violence, sexual abuse, emotional neglect, emotional cruelty, and bullying) (see Supplement Section 2 for details of questionnaire items and timepoints). Each trauma type was binarised into 0 or 1, based on whether an individual reported exposure to the specific trauma type at any age.

Missing data were imputed for the whole ALSPAC cohort (*n* = 15,645) on the six types of childhood psychological trauma and the pre- and perinatal risk factor variables available in the ALSPAC dataset through multiple imputation by chained equations (MICE), iterated through 25 cycles (Stata v17). Little’s test determined if data were missing completely at random (MCAR). Logistic regressions determined whether missingness for (i) pre/perinatal risk items and (ii) trauma type items were associated with demographic variables, and significant variables (which also significantly related to PEs group) were included in the MICE models as auxiliary variables.

The two composite weighted variables (cumulative childhood trauma, cumulative pre/perinatal risk) were generated post imputation. The natural logarithm of the odds ratio (OR^ln^) for the association between a specific risk factor and psychosis was used to weight the risk factor items (risk factor multiplied by OR^ln^), according to published protocols [60], using odds ratios for pre/perinatal risk factors reported in a recent meta-analysis [3] and a recent study of psychological trauma in the ALSPAC dataset [6]. To generate cumulative scores, weighted risk factors were summed and then divided by the number of risk factors (16 for pre and perinatal risk, 6 for psychological trauma). Sensitivity analyses were conducted on non-weighted summary scores.

### MRI

MRI data were acquired at the Cardiff University Brain Imaging Centre (CUBRIC) on a 3T scanner (Signa HDx;GE Medical Systems) using an 8-channel head coil for radiofrequency reception. A high-resolution, 3D fast spoiled gradient-echo (FSPGR) T1-weighted isotropic image was oriented to the AC–PC line (TR = 7.8 ms, TE = 3 ms, inversion time = 450 ms, flip angle = 20°, field of view = 256 mm × 256 mm × 192 mm, 1 mm isotropic resolution) to assess grey matter volume.

### Voxel-based morphometry (VBM)

We conducted image preprocessing including segmentation and DARTEL normalisation using the Computational Anatomy Toolbox (CAT12, http://dbm.neuro.uni-jena.de/cat12/) for Statistical Parametric Mapping (SPM 12). ‘Modulated’ images corrected for non-linear deformations were used for analyses. Spatial smoothing used a Gaussian kernel of 8 mm full width at half maximum using SPM 12 standard routines. Voxel-wise comparison of modulated T1-segmented grey matter images was performed using several general linear models. In separate models, we examined the effect of (1) PEs (classified on a 4-point ordinal scale: no PEs < suspected PEs < definite PEs < clinical disorder), (2) cumulative psychological trauma and (3) cumulative pre/perinatal risk. To determine if cumulative risk has differential effects on volume depending on PEs group, we used separate models to examine (1) the interaction between PEs and cumulative psychological trauma and (2) the interaction between PEs and cumulative pre/perinatal risk. Age, sex, antipsychotic medication and total intracranial volume (TIV) were included as covariates in all models. In addition, interaction models included their respective main effects (cumulative risk score and PE). Thresholds were set at *p* < 0.001 uncorrected at the voxel level, together with a family-wise error (FWE) correction for multiple comparisons at pFWE < 0.05 at the cluster level.

### Statistical analysis

Inferential statistics on associations between demographic variables and PEs group (no PEs, suspected PEs, definite PEs, psychotic disorder) were conducted in Stata (v17) using Chi square (χ^2^) or linear regression. Linear regression determined whether (i) cumulative psychological trauma and (ii) cumulative pre/perinatal risk associated with PEs group. Significant cluster volumes from VBM analyses were extracted using the SPM toolbox ‘MarsBaR’ and were plotted using the R package ‘ggplot2’. Pairwise comparisons for cluster volumes associated with PEs group were examined using Dunnett’s post-hoc tests in R. For significant interactions, linear regressions determined if slopes significantly differed from zero for each group separately.

## Results

MRI data were available for 434 participants from the two ALSPAC imaging studies. 21 participants did not receive PEs assessments and were excluded. Analyses were conducted on 413 participants (*n* = 275 participants without PEs, *n* = 49 with suspected PEs, *n* = 53 with definite PEs and *n* = 36 with psychotic disorder). Sample demographics are reported in Table 1. PEs groups were associated with slightly younger participants at the time of scanning (*p* < 0.001), a trend for lower TIV (*p* = 0.03) and a higher proportion of female participants (*p* = 0.07). See Supplementary Table 2 for cumulative psychological trauma and pre/perinatal risk factor missingness. Little’s test found that data were not MCAR (χ2 distance = 4355, df = 1464, *p* < 0.001). Missingness for the 16 pre/perinatal risk items was associated with maternal SES, maternal education and maternal smoking. Missingness for the 6 psychological trauma type items (physical cruelty, domestic violence, sexual abuse, emotional neglect, emotional cruelty, and bullying) was associated with sex, PEs group, maternal SES, maternal education, maternal smoking and mother diagnosed depression. Variables associated with missingness were also associated with PEs group (Supplementary Table 3).

**Table 1:** Participant demographics, and association between psychotic experiences (PEs) and environmental risk factors (cumulative psychological trauma and cumulative pre/perinatal risk factors, weighted for their association with psychosis).

Variable	PEs				Inferential Statistics
	None	Suspected	Definite	Psychotic Disorder	
Total	275	49	53	36	
Antipsychotic medication	0	0	0	6	
Sex: female	156 (62%)	33 (13%)	33 (13%)	28 (11%)	χ2 = 7.167, *p* = 0.07
Sex: male	119 (73%)	16 (10%)	20 (12%)	8 (5%)	
Age: years	21.53 (1.46)	20.78 (1.05)	20.17 (1.01)	20.11 (0.92)	Coefficient −0.27 (95%CI −0.34 to −0.21) *p* < 0.001
Total intracranial volume	1454.88 (139.06)	1428.88 (145.80)	1454.83 (151.09)	1386.04 (162.68)	Coefficient −0.0007 (95%CI −0.00141 to −0.00006) *p* = 0.03
Cumulative psychological trauma	0.10 (0.10)	0.15 (0.13)	0.17 (0.16)	0.18 (0.15)	Coefficient 2.16 (95%CI 1.38 to 2.93) *p* < 0.001
Cumulative pre/perinatal risk	0.039 (0.037)	0.035 (0.030)	0.038 (0.038)	0.046 (0.045)	Coefficient 0.68 (95%CI −1.98 to 3.33) *p* = 0.62

Inferential statistics used Chi square (χ2) or linear regression. Standard deviation or % presented in brackets. Trauma types are: physical cruelty, domestic violence, sexual abuse, emotional neglect, emotional cruelty, and bullying. See Supplementary Table 1 for the 16 pre/perinatal risk factors for psychosis.

### Association between psychotic experiences and environmental risk factors

Cumulative psychological trauma was associated with PEs (Coefficient 2.16 (95%CI 1.38 to 2.93) *p* < 0.001, Table 1). Cumulative pre/perinatal risk was not associated with PEs (Table 1). Cumulative pre/perinatal risk was not associated with cumulative psychological trauma (Coefficient 0.68 (95%CI −1.98 to 3.33) *p* = 0.62).

### Brain structure and psychotic experiences

**Fig. 1:**
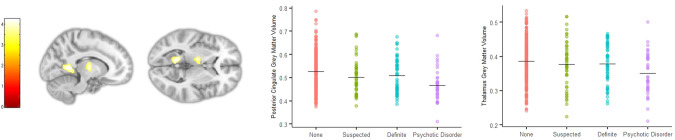
Psychotic experiences (PEs) were associated with smaller grey matter volume in the left posterior cingulate and left thalamus. Plots show mean and individual posterior cingulate and thalamus grey matter volume for each PEs group.

PEs were associated with smaller grey matter volume in the left posterior cingulate (Fig. 1, *p*FWE < 0.001; −12, −53, 5; *Z* = 4.19; 687 voxels) and the left thalamus (*p*FWE = 0.006; −15, −11, 9; *Z* = 3.91; 445 voxels). Post-hoc tests found significantly smaller posterior cingulate and thalamus volumes in psychotic disorder cases compared to controls (*t* = −4.48, *p* < 0.001 and *t* = −3.329, *p* = 0.003 respectively). Thalamus volume did not significantly differ between controls and suspected (*t* = −0.95, *p* = 0.71) or definite PEs cases (*t* = −0.78, *p* = 0.82). Suspected and definite PEs cases showed intermediate posterior cingulate volumes between those of controls and those with psychotic disorder, which did not significantly differ from controls (*t* = −2.11, *p* = 0.10 and *t* = −1.45, *p* = 0.38).

### Brain structure and environmental risk factors

**Fig. 2:**
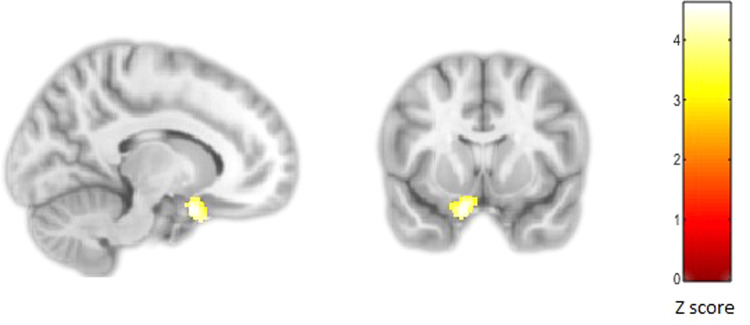
Cumulative pre/perinatal risk was associated with smaller volume in the left subgenual cingulate in all participants (PEs and controls). Cumulative pre/perinatal risk is the summed score of 16 pre/perinatal risk factors weighted for their association with psychosis.

**Fig. 3:**
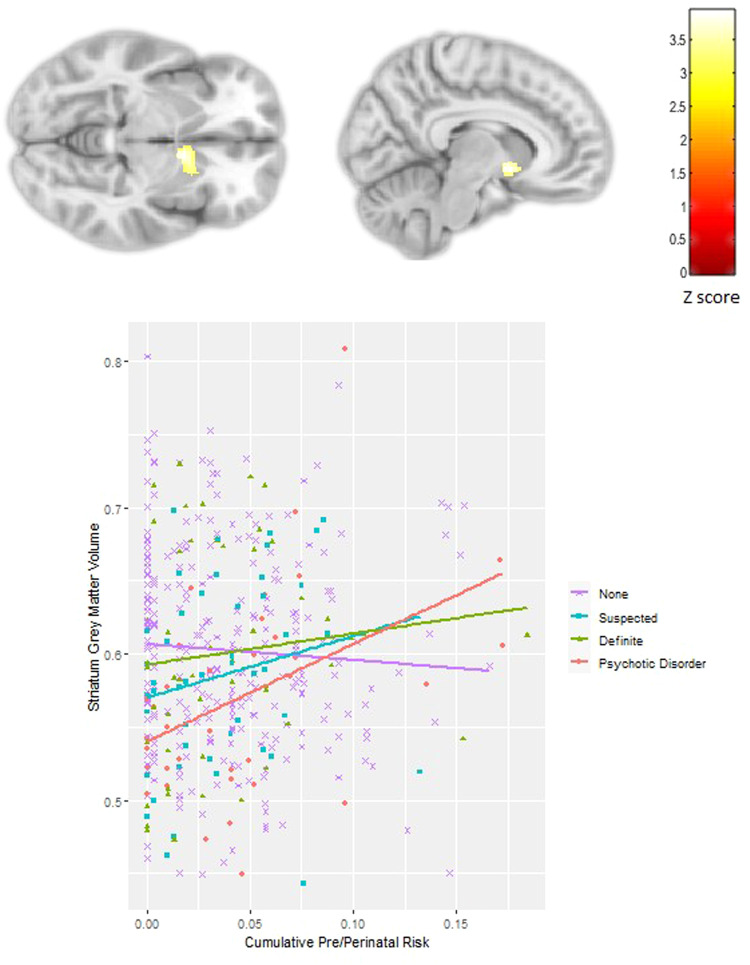
Significant interaction between psychotic experiences (PEs) and cumulative pre/perinatal risk (16 risk factors weighted for their association with psychosis) in the right nucleus accumbens, caudate and putamen (striatum). Cumulative pre/perinatal risk was associated with larger striatal volume in those with psychotic disorder.

**Fig. 4:**
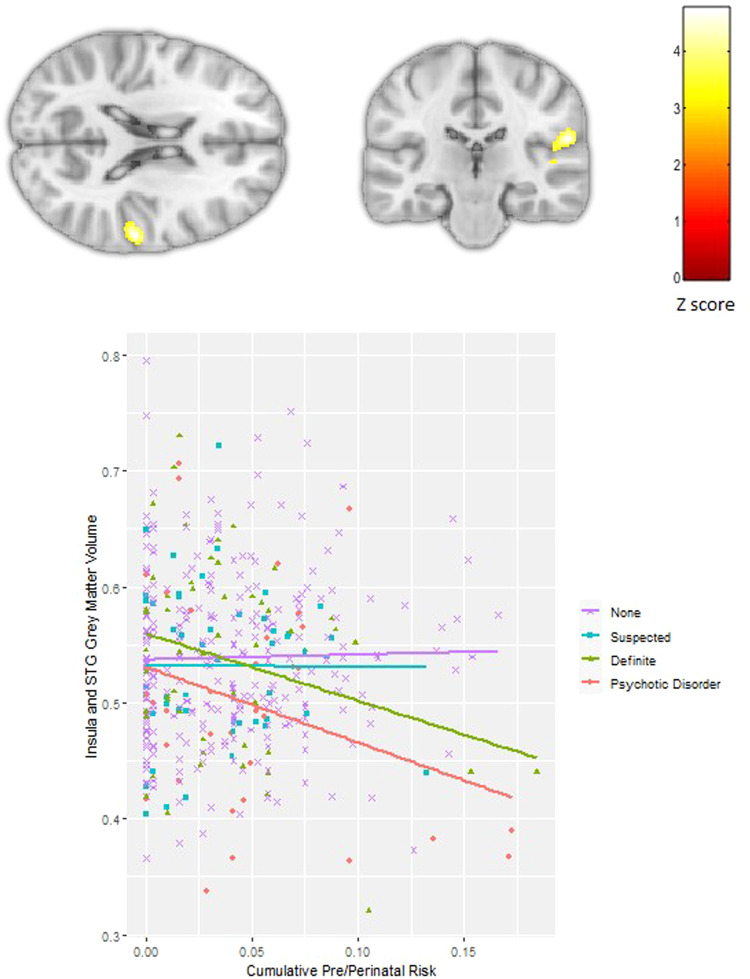
Significant interaction between psychotic experiences (PEs) and cumulative pre/perinatal risks (16 risk factors weighted for their association with psychosis) in the right insula, supramarginal gyrus and superior temporal gyrus (STG). Cumulative pre/perinatal risk was associated with smaller volume in those with definite PEs and psychotic disorder cases.

Cumulative pre/perinatal risk was associated with smaller left subgenual cingulate volume extending into the inferior frontal gyrus in all subjects (*p*FWE < 0.001; −12, 9, −24; *Z* = 4.54; 788 voxels, Fig. 2). There was a significant interaction between PEs and cumulative pre/perinatal risk in the right nucleus accumbens, caudate and putamen (striatum) (*p*FWE = 0.04; 9, 6, −6; *Z* = 3.89; 306 voxels, Fig. 3) and the right insula extending into the supramarginal gyrus and superior temporal gyrus (*p*FWE = 0.002; 59, −24, 20; *Z* = 4.79; 521 voxels, Fig. 4). Higher cumulative pre/perinatal risk was associated with smaller insula, supramarginal gyrus and superior temporal gyrus volumes in those definite PEs and psychotic disorder (trend for significant difference in slope from zero for definite PEs: *t* = −1.97, *p* = 0.06 and psychotic disorder: *t* = −1.91, *p* = 0.07). Higher cumulative pre/perinatal risk was associated with larger striatal volume in psychotic disorder cases (significant difference in slope from zero: *t* = 2.71, *p* = 0.01).

**Fig. 5:**
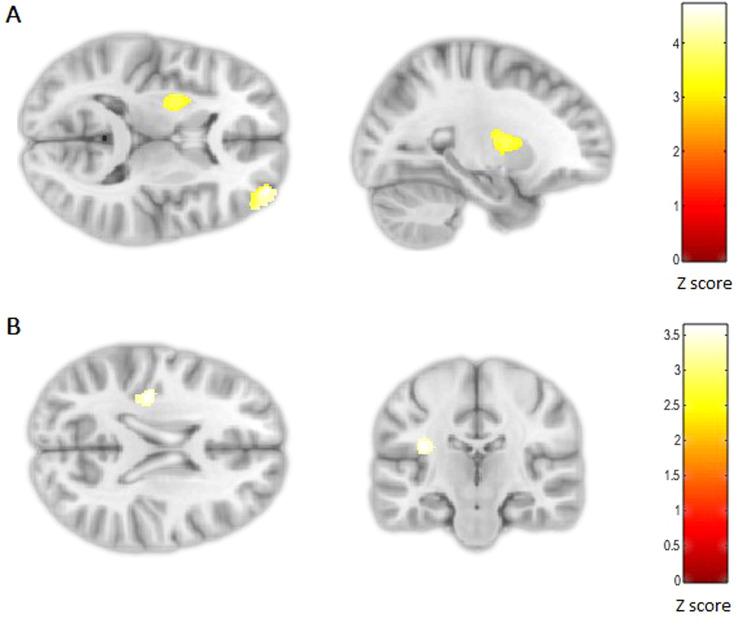
Cumulative psychological trauma was associated with larger volumes in the striatum and middle frontal gyrus, and smaller insula volume in all participants (PEs and controls). Cumulative psychological trauma (6 trauma types weighted for their association with PEs) was associated with larger volume in the left striatum and right middle frontal gyrus (**A**: top panel) and smaller left insula volume (**B**: bottom panel) in all participants.

Cumulative psychological trauma was associated with larger volumes in the left putamen (dorsal striatum) (Fig. 5, *p*FWE = 0.002; −24, −2, 11; *Z* = 3.65; 523 voxels) and right middle frontal gyrus (*p*FWE < 0.001; 38, 59, 14; *Z* = 4.63; 720 voxels), and smaller volume in the left insula (*p*FWE = 0.03; −35, −21, 21; *Z* = 3.60; 335 voxels). There was no significant interaction between group and cumulative psychological trauma. Sensitivity analyses on non-weighted summary scores for cumulative psychological trauma (score 0–6) and cumulative pre/perinatal risk (score 0–16) are reported in the Supplement, finding the same results with the exception of the insula.

## Discussion

This study utilised the ALSPAC birth cohort which features rich exposure data on multiple pre- and perinatal risk factors as well as longitudinal measures of multiple types of childhood psychological trauma, to assess their impact on brain volume and on PEs. Our initial finding showed PEs status in young people was associated with smaller grey matter volume in the posterior cingulate and thalamus, key regions implicated in schizophrenia [61, 62]. Thalamic volume alterations were specific to psychotic disorder cases, whereas reductions in posterior cingulate volume related to the severity of PEs; with intermediate volumes in suspected and definite PEs groups, and the smallest volume in psychotic disorder. This lends support for a continuum view of the pathophysiology of schizophrenia.

Our results suggest that diverse environmental risk factors affect similar brain pathways, as both cumulative psychological trauma and pre/perinatal risk factors were associated with larger striatum and smaller insula volumes. The effect of cumulative pre/perinatal risk on striatum and insula volume was only apparent in those with definite PEs or psychotic disorder, whereas cumulative psychological trauma was associated with these brain changes in all participants regardless of PEs status. Both the insula and striatum are altered in schizophrenia [63–66], and so it is conceivable that these changes could increase an individual’s propensity to develop schizophrenia.

Prefrontal and striatum volume increases in those exposed to early psychological trauma may be adaptive, as exposure to adverse environments during childhood lead to increased vigilance to threatening stimuli [67] and increased frontal and striatal dopamine activity [68–70]. Increased frontal and basal ganglia volumes have been previously reported in transdiagnostic samples [43], healthy individuals and patients with schizophrenia exposed to childhood trauma [38, 40], and may reflect premature maturation of these regions [38]. Striatal volume alterations associated with cumulative pre/perinatal risk varied according to PEs group. Typically, widespread reductions in cortical grey matter and subcortical volumes [14, 15, 17, 18, 22–26] are associated with very preterm birth (often defined as <32 weeks gestational age). This pattern was seen in controls, whereas in those with psychotic disorder, striatal volume increased with pre/perinatal risk load. The large deviation in striatal volume in psychotic disorder (smaller or larger volume than controls depending on prenatal exposure) may reflect greater variance in regional brain volumes in schizophrenia compared to controls, as shown in meta-analyses [71].

Both risk factor types were also associated with smaller insula volume. Significant clusters associated with cumulative psychological trauma were localised to the insula, whereas a larger area encompassing the insula, supramarginal gyrus and superior temporal gyrus was associated with pre/perinatal risk. Such volume alterations may increase an individual’s vulnerability to developing psychosis, as previous studies report smaller insula and superior temporal gyrus volumes in schizophrenia, schizotypy and subjects at high risk for psychosis [65, 72, 73], and those that transition to psychosis [74–76]. The insula generates prediction errors by comparing expected interoceptive states with external sensory input [77], and its disruption may weaken discrimination between self-generated and external information. The superior temporal gyrus is associated with language processing and thus may contribute to the experience of auditory hallucinations in those with PEs and psychotic disorders [78].

Pre/perinatal risk shows effects specific to those with PEs (insula and striatum), but also effects in all participants (subgenual cingulate). This is reflected by the literature, where preterm birth has an exacerbated effect on the brain in schizophrenia patients compared to controls [27, 29]. The insula and subgenual cingulate form the salience network, which is shown to be disrupted in schizophrenia [79]. Alterations in both regions, as seen in definite PEs and psychotic disorder cases, may have a compounding effect and increase an individual’s vulnerability to psychosis. Similar to our findings, a recent cohort study found reduced anterior cingulate volume with obstetric adversity [38], whilst another reported associations between obstetric complications and reduced orbitofrontal and insula volume, as well as reduced middle temporal and inferior parietal cortex [80].

Effects of childhood trauma on the brain were seen in all participants and were not specific to individuals at a higher risk of developing schizophrenia. This is largely consistent with previous findings which report similar brain volume alterations in healthy individuals and groups at a high risk of schizophrenia exposed to trauma [11, 12, 39, 42–44]. These studies generally find lower volumes rather than higher volumes as detected in the present study, principally in amygdala, hippocampus [31, 32, 34] and frontal regions [81], although recent studies describe a pattern of both smaller and larger brain volumes associated with sexual abuse or childhood trauma, mapping onto our findings of increased striatal and frontal volumes [38, 43]. Moreover, a meta-analysis reports that childhood maltreatment is associated with increases in frontal cortex (Brodmann area 10) and reductions in insula volume, alongside reductions in other regions including the amygdala, hippocampus and temporal gyri [33].

Interaction effects like those described for pre/perinatal risk, whereby insula and striatal volume alterations were only seen in those with PE, can result from type I errors due to a reduction in detectable group effect sizes and so should be interpreted with caution. Consequently, sex differences were not examined due to limitations in power, although these have been previously reported [32, 34, 43], although not by all studies [82]. Psychological trauma was associated with PEs, however pre/perinatal risk did not show an association. Reported odds ratios for the association between pre/perinatal risk and schizophrenia are modest [3] and may be weaker for subclinical populations, as previous reports on the ALSPAC study show that associations are less strong when including suspected PEs as part of the outcome [4]. This precluded attempts to run a mediation analysis, in addition to the lack of overlap in significant brain regions associated with PEs and regions associated with environmental risk factors. Instead, effects of environmental adversity on the brain may ‘accumulate’, and additional pathophysiological mechanisms may be required to develop psychosis.

While this study used a whole brain analysis approach which has the advantage of not biasing the study to a particular brain region, we may have had less power to examine changes in smaller subcortical structures such as the amygdala and hippocampus, which have previously been implicated in trauma research [83]. We did not incorporate data on the timing of childhood trauma which may incur differential effects on the brain [84], however, a significant proportion of the sample experienced trauma both pre- and post-puberty, making it difficult to disentangle these effects. Cumulative exposure variables were derived using imputation to estimate missing data. Missing data was minimal for psychological trauma types, however rates of missing data were higher for pre/perinatal risk factors (30% for 11 items, 20% for 2 items). To improve imputation estimates, auxiliary variables associated with missingness were added to the model, making the ‘missing at random’ assumption more plausible [85]. This study focuses on the cumulative influence of environmental risk factors and does not inform on the effect of individual risk factors. Strengths of our study include the use of prospective measurements of childhood trauma at multiple timepoints and rich birth record data on a large number of pre- and perinatal risk factors. Our analyses used a whole brain approach and so identified additional brain regions to those typically explored by ROI analyses.

Volume alterations in the insula and striatum may represent a common pathway affected by different environmental risk factors for psychosis, as both pre/perinatal risk and psychological trauma were independently associated with increased striatal and reduced insula volumes, despite these risk factors not being correlated in our sample. Both risk factors affect brain networks implicated in schizophrenia: cumulative pre/perinatal risk impacts the salience network, namely the subgenual cingulate and insula, and cumulative psychological trauma impacts fronto-striatal networks related to threat processing. Further research is needed to identify beneficial environmental interventions to inform future public health initiatives.

### Supplementary information

SUPPLEMENTAL MATERIAL
