# Supplementary material for: The impact of cumulative obstetric complications and childhood trauma on brain volume in young people with psychotic experiences
Source: Mol Psychiatry. 2023 Oct 30;28(9):3688–97. doi: 10.1038/s41380-023-02295-6 (PMC10730393; doi:10.1038/s41380-023-02295-6)
Supplement: Supplementary file 1 — SUPPLEMENTAL MATERIAL [file 41380_2023_2295_MOESM1_ESM.docx]

Contents

[1. Methods: Cumulative Pre- and Perinatal Risk Score (Table 1) 1](#_Toc144463822)

[2. Methods: Six Trauma Type Measures 2](#_Toc144463823)

[Table 2: Missingness 3](#_Toc144463824)

[Table 3: Association between Missingness and Demographic Variables 4](#_Toc144463825)

[Sensitivity Analyses: Brain Structure and Environmental Risk Factors 4](#_Toc144463826)

[References 4](#_Toc144463827)

## Methods: Cumulative Pre- and Perinatal Risk Score (Table 1)

Prenatal and perinatal risk factors were identified from a recent review(1). Biological risk factors showing an association with psychosis at P<0.06 were included in the Cumulative Prenatal Risk Score. 24 risk factors were available from the ALSPAC cohort (Factors not available: Congenital malformation, Toxoplasma, Any famine or nutritional deficit, Famine, Ruptured membranes). A factor was not included in the cumulative exposure variable if any of the case or control groups contained zero participants (Maternal age at delivery <20), or if >40% of the sample were exposed to a pre/peri-natal factor (Unspecified obstetric complications, Unspecified maternal infections, Definite obstetric complications). Variables indirectly linked to biological risk factors were not included (winter to spring birth, 3 pregnancies or more, suboptimal number of antenatal visits, stress during pregnancy).

|  | **OR (95% CI)** | **p value** |  |
| --- | --- | --- | --- |
| **Included Risk Factors:** |  |  |  |
| <37 weeks (premature birth) | 1·35 (1·12–1·62) | 0·0016 |  |
| Birthweight <2500g | 1·53 (1·31–1·78) | <0·0001 |  |
| Birth length <49 cm | 1·17 (1·05–1·32) | 0·057 |  |
| Head circumference <32 cm | 1·37 (0·99–1·91) | 0·057 |  |
| Asphyxic state | 1·93 (1·30–2·88) | 0·0012 |  |
| Maternal age at delivery 30–34yrs | 1·05 (1·01–1·09) | 0·019 |  |
| Paternal age <20yrs | 1·31 (1·17–1·46) | <0·0001 |  |
| Paternal age ≥35yrs | 1·28 (1·06–1·55) | 0·012 |  |
| Herpes simplex type 2 | 1·35 (1·16–1·58) | 0·0002 |  |
| Maternal hypertension | 1·40 (1·10–1·78) | 0·0058 |  |
| Pre-eclampsia or toxaemia | 1·32 (0·99–1·76) | 0·059 |  |
| Hypoxia | 1·63 (1·11–2·40) | 0·014 |  |
| Premature rupture | 2·29 (1·38–3·80) | 0·0013 |  |
| Polyhydramnios | 3·05 (1·15–8·06) | 0·025 |  |
| Blood loss during pregnancy | 1·54 (1·06–2·25) | 0·023 |  |
| Rhesus-associated factors and incompatibility | 1·42 (1·00–2·01) | 0·051 |  |

Table 1. Risk factors included in the prenatal risk summary score. Odds Ratio (OR), confidence intervals (95% CI) and P values are extracted from the review by Davies et al., 2002

## Methods: Six Trauma Type Measures

Responses to a range of questionnaires from children and caregivers regarding trauma exposures, including items taken from the validated Child Abuse Questionnaire and the Sexual Experiences Survey, were used to derive measures of exposure to six trauma types. Measures were collected prospectively from ages 0 to 17 years old and supplemented by data collected at aged 22 that referred to traumatic exposures that occurred in childhood (before 11) and adolescence (11-17).

Questions used to derive the trauma-type categories:

1. **Physical abuse:** The following were used to derive the measure: 19 questions from parents at 0-5 years, 13 questions from parents and 5 questions from children at 5-11 years and 5 questions from parents and 11 questions from children at 11-17 years. Of the questions completed by children, 5 questions at 5-10.9 years and 2 questions at 11-17 were reported at 22 years referring to these timepoints retrospectively.

Eg ‘In the last year, has someone hit, kicked, punched or attacked you with the intention of really hurting you?’

1. **Sexual abuse**: A total of 3 questions at 0-5 by parents, 4 questions from parents at 5-11, 4 questions from children at 5-11 and 6 questions from children at 11-17. Of the questions completed by children, 2 questions at 5- 10.9 years and all 6 questions at 11-17 were reported at 22 years referring to these timepoints retrospectively.

Eg ‘Has an adult or older child forced, or attempted to force, you into sexual activity?’

1. **Emotional abuse:** The following were used to derive the measure: 18 questions by parents at 0-5, 13 questions from parents and 4 questions from children at 5-11 years and 4 questions from parents and 3 questions from children at 11-17 years. Of the questions completed by children, 3 questions at 5-10.9 years and 3 questions at 11-17 were reported at 22 years referring to these timepoints retrospectively.

Eg ‘How often has an adult in the family said hurtful or insulting things to you?’ (‘often’ or ‘very often’ classified as emotional abuse).

1. **Emotional neglect**: There were no questions available for a measure between 0-5 years, 2 questions between 5-11 and 5 questions between 11-17, all completed by children, were used to derive the measure.

Eg ‘How often does a caregiver know where you were going, when you went out, in the last year?’ (‘never’ classified as emotional neglect).

1. **Bullying**: A total of 2 questions at 0-5 by parents, 3 questions from parents and 4 questions from children at 5-11, 2 questions by parents and 3 questions by children at 11-17.

Eg How often have you been threatened or blackmailed?’ (more than four times in the last six months classified as bullying).

1. **Domestic violence:** A total of 11 questions at 0-4.9 8 questions at 5-10.9 and 4 questions at 11-17 all reported by parents were used to derive this measure.

Eg ‘Has your partner been physically cruel towards you in the past year?’ (asked of parents; domestic violence).

For more details, see Croft J, Heron J, Teufel C, Cannon M, Wolke D, Thompson A, et al. Association of Trauma Type, Age of Exposure, and Frequency in Childhood and Adolescence With Psychotic Experiences in Early Adulthood. JAMA Psychiatry. 2019;76:79

## Table 2: Missingness

| **Variable** | **Number missing** | **% missing** |
| --- | --- | --- |
| **Cumulative Trauma** | | |
| Emotional cruelty | 12 | 3% |
| Physical cruelty | 12 | 3% |
| Domestic violence | 12 | 3% |
| Sexual abuse | 16 | 4% |
| Emotional neglect | 17 | 4% |
| Bullying | 16 | 4% |
| **Pre/Perinatal score** | | |
| Birthweight <2500g | 19 | 5% |
| Paternal age <20 | 132 | 32% |
| Paternal age ≥35 | 132 | 32% |
| Herpes simplex type 2 | 132 | 32% |
| Maternal age at delivery 30–34 | 22 | 5% |
| Asphyxic state | 132 | 32% |
| Premature Rupture | 146 | 35% |
| <37 weeks (premature birth) | 133 | 32% |
| Maternal hypertension | 142 | 34% |
| Hypoxia (Resuscitated at birth) | 149 | 36% |
| Blood loss during pregnancy (postpartum haemorrhage) | 144 | 35% |
| Polyhydramnios | 132 | 32% |
| Rhesus factors (Rh antibodies noted during pregnancy before onset of labour and/or Anti D given during pregnancy before onset of labour) | 132 | 32% |
| Birth length <49 cm | 93 | 23% |
| Head circumference <32 cm | 89 | 22% |
| Pre-eclampsia: 2 occasions with 1+ proteinuria | 15 | 4% |

Table 2. Missing data for variables that make up the summary scores for 1) cumulative trauma and 2) cumulative pre and perinatal risk. Missingness for the imaging sample is shown; multiple imputation was performed on the whole ALSPAC sample.

## Table 3: Association between Missingness and Demographic Variables

|  | Pre/perinatal risk items | | Trauma type items | | PE group |  |
| --- | --- | --- | --- | --- | --- | --- |
|  | z value | p value | z value | p value | z value | p value |
| PE group | -0.75 | 0.454 | 2.27 | **0.023** |  |  |
| Sex | -0.26 | 0.793 | -4.09 | **<0.001** | 3.55 | **<0.001** |
| Maternal education | -12.48 | **<0.001** | -23.33 | **<0.001** | -5.07 | **<0.001** |
| Maternal SES | 5.47 | **<0.001** | 9.19 | **<0.001** | 2.98 | **0.003** |
| Maternal alcohol consumption | -0.37 | 0.71 | -0.95 | 0.342 |  |  |
| Maternal smoking | 8.55 | **<0.001** | 18.6 | **<0.001** | 6.76 | **<0.001** |
| Mother diagnosed depression | -0.12 | 0.904 | 8.08 | **<0.001** | 1.79 | 0.074 |
| Mother diagnosed schizophrenia | -0.24 | 0.814 | 1.64 | 0.100 |  |  |

Table 3. Logistic and ordinal regression examining association between demographic variables and i) missingness for pre/perinatal risk items, ii) missingness for psychological trauma type items, and iii) PE group.

## Sensitivity Analyses: Brain Structure and Environmental Risk Factors

Sensitivity analyses examined cumulative psychological trauma and cumulative pre/perinatal risk, without weighting individual risk factors according to their association with psychosis. Cumulative pre/perinatal risk was calculated by summing 16 binarised (exposed/not exposed) pre/perinatal risk factors to create a score from 0 to 16. Cumulative psychological trauma was calculated by summing 6 binary psychological trauma variables (physical cruelty, domestic violence, sexual abuse, emotional neglect, emotional cruelty, and bullying). If an individual reported exposure to a trauma type at any age, this contributed a score of 1 to the summary score, to create a score from 0 to 6.

Cumulative pre/perinatal risk was associated with smaller left subgenual cingulate volume extending into the inferior frontal gyrus in all subjects (*p*FWE=0.003; -12, 9, -23; *Z*=4.46; 500 voxels). There was a significant interaction between PEs and cumulative pre/perinatal risk in the right nucleus accumbens, caudate and putamen (striatum) (*p*FWE<0.001; 11, 6, -6; *Z*=4.03; 813 voxels). Higher cumulative pre/perinatal risk was associated with larger striatal volume in psychotic disorder cases (significant difference in slope from zero: *t*=2.37, *p*=0.02).

Cumulative psychological trauma was associated with larger volumes in the left putamen (dorsal striatum) (*p*FWE=0.002; -24, -5, 9; *Z*=3.94; 731 voxels) and right middle frontal gyrus (*p*FWE<0.001; 38, 57, 14; *Z*=4.78; 758 voxels). There was a significant interaction between PEs and cumulative psychological trauma in the left cerebellum crus-I/II (*p*FWE=0.02; -26, -75, -33; *Z*=3.59; 345 voxels). Higher cumulative psychological trauma was associated with larger cerebellum crus-I/II volume in psychotic disorder cases (significant difference in slope from zero: *t*=2.54, *p*=0.02).

## References

1. Davies C, Segre G, Estradé A, Radua J, De Micheli A, Provenzani U, et al. Prenatal and perinatal risk and protective factors for psychosis: a systematic review and meta-analysis. The Lancet Psychiatry. 2020 May 1;7(5):399–410.
